# Carbolic Acid

**Published:** 1869-07-01

**Authors:** 


### *Carbolic Acid.*

We think it necessary to put our readers on their guard against an incautious use of carbolic acid. It seems to be forgotten, sometimes, that this substance exercises a powerfully destructive action upon animal tissues, and that it is, in fact, a very strong caustic when concentrated. There is no doubt that many serious accidents have recently occurred from surgeons not being aware of the properties of the remedy they use so freely. It must also be remembered that the direct application of carbolic acid, even in a diluted form, to a granulating surface, will often delay cicatrization, and tend to promote suppuration; whereas, if it is employed at a distance from the wound, it will tend to diminish the formation of pus. There is, moreover, a good deal of evidence to show that it tends to stimulate the circulation through the smaller vessels, and thus gives rise to hæmorrhagic oozing from recently cut surfaces, preventing their primary adhesion. If, however, it be properly applied, in a diluted form, to the wound itself, and in some permanent and non-volatile form to the external parts, it will be found to have a powerful influence in retarding and diminishing suppuration.—*Medical News.*

